# Supplementary material for: A Cross-Sectional Description of Parental Perceptions and Practices Related to Risky Play and Independent Mobility in Children: The New Zealand State of Play Survey
Source: Int J Environ Res Public Health. 2019 Jan 17;16(2):262. doi: 10.3390/ijerph16020262 (PMC6352286; doi:10.3390/ijerph16020262)
Supplement: Supplementary file 1 [file ijerph-16-00262-s001.zip › Jelleyman AF 2 SoP survey.docx]

**SECTION 1**

**Q1**

Household Structure

How many dependent children younger than 19 years of age currently live with you?

|  |
| --- |

**Q2**

Please provide the gender and age of each of the dependent children younger than 19 years that currently live with you.

Child 1 gender:

1. Male
2. Female

Child 1 age:

1. Less than 1 year of age
2. 1 year old
3. 2 years old
4. 3 years old
5. 4 years old
6. 5 years old
7. 6 years old
8. 7 years old
9. 8 years old
10. 9 years old
11. 10 years old
12. 11 years old
13. 12 years old
14. 13 years old
15. 14 years old
16. 15 years old
17. 16 years old
18. 17 years old
19. 18 years old

Child 2 gender:

1. Male
2. Female

Child 2 age:

1. Less than 1 year of age
2. 1 year old
3. 2 years old
4. 3 years old
5. 4 years old
6. 5 years old
7. 6 years old
8. 7 years old
9. 8 years old
10. 9 years old
11. 10 years old
12. 11 years old
13. 12 years old
14. 13 years old
15. 14 years old
16. 15 years old
17. 16 years old
18. 17 years old
19. 18 years old

Child 3 gender:

1. Male
2. Female

Child 3 age:

1. Less than 1 year of age
2. 1 year old
3. 2 years old
4. 3 years old
5. 4 years old
6. 5 years old
7. 6 years old
8. 7 years old
9. 8 years old
10. 9 years old
11. 10 years old
12. 11 years old
13. 12 years old
14. 13 years old
15. 14 years old
16. 15 years old
17. 16 years old
18. 17 years old
19. 18 years old

Child 4 gender:

1. Male
2. Female

Child 4 age:

1. Less than 1 year of age
2. 1 year old
3. 2 years old
4. 3 years old
5. 4 years old
6. 5 years old
7. 6 years old
8. 7 years old
9. 8 years old
10. 9 years old
11. 10 years old
12. 11 years old
13. 12 years old
14. 13 years old
15. 14 years old
16. 15 years old
17. 16 years old
18. 17 years old
19. 18 years old

Child 5 gender:

1. Male
2. Female

Child 5 age:

1. Less than 1 year of age
2. 1 year old
3. 2 years old
4. 3 years old
5. 4 years old
6. 5 years old
7. 6 years old
8. 7 years old
9. 8 years old
10. 9 years old
11. 10 years old
12. 11 years old
13. 12 years old
14. 13 years old
15. 14 years old
16. 15 years old
17. 16 years old
18. 17 years old
19. 18 years old

Child 6 gender:

1. Male
2. Female

Child 6 age:

1. Less than 1 year of age
2. 1 year old
3. 2 years old
4. 3 years old
5. 4 years old
6. 5 years old
7. 6 years old
8. 7 years old
9. 8 years old
10. 9 years old
11. 10 years old
12. 11 years old
13. 12 years old
14. 13 years old
15. 14 years old
16. 15 years old
17. 16 years old
18. 17 years old
19. 18 years old

Child 7 gender:

1. Male
2. Female

Child 7 age:

1. Less than 1 year of age
2. 1 year old
3. 2 years old
4. 3 years old
5. 4 years old
6. 5 years old
7. 6 years old
8. 7 years old
9. 8 years old
10. 9 years old
11. 10 years old
12. 11 years old
13. 12 years old
14. 13 years old
15. 14 years old
16. 15 years old
17. 16 years old
18. 17 years old
19. 18 years old

Child 8 gender:

1. Male
2. Female

Child 8 age:

1. Less than 1 year of age
2. 1 year old
3. 2 years old
4. 3 years old
5. 4 years old
6. 5 years old
7. 6 years old
8. 7 years old
9. 8 years old
10. 9 years old
11. 10 years old
12. 11 years old
13. 12 years old
14. 13 years old
15. 14 years old
16. 15 years old
17. 16 years old
18. 17 years old
19. 18 years old

**Q3**

Which one of these statements is true about your current legal marital / civil union status?

1. Single and never married / in a civil union
2. Married / in a civil union or living with a partner
3. Permanently separated or divorced
4. Widowed
5. Other ____________________________________________________

**Q4**

How many adults over the age of 18 years currently live in your household (including yourself)?

1. One adult
2. Two adults
3. Three adults
4. Four adults
5. More than four adults

**Q5**

Perceptions of Play

At what age (if at all) should children first be allowed to:

|  | Younger than 2 years of age | 2-3 years of age | 4-5 years of age | 6-7 years of age | 8-9 years of age | 10-11 years of age | 12-13 years of age | 14-15 years of age | 16-18 years of age | Children should not be allowed |
| --- | --- | --- | --- | --- | --- | --- | --- | --- | --- | --- |
| Climb trees at home or in their local park/recreation areas? | ❏ | ❏ | ❏ | ❏ | ❏ | ❏ | ❏ | ❏ | ❏ | ❏ |
| Climb trees at their early childhood centre / school? | ❏ | ❏ | ❏ | ❏ | ❏ | ❏ | ❏ | ❏ | ❏ | ❏ |
| Engage in rough-and-tumble games (e.g., wrestling, bullrush) at home? | ❏ | ❏ | ❏ | ❏ | ❏ | ❏ | ❏ | ❏ | ❏ | ❏ |
| Engage in rough-and-tumble games (e.g., wrestling, bullrush) at their early childhood centre / school? | ❏ | ❏ | ❏ | ❏ | ❏ | ❏ | ❏ | ❏ | ❏ | ❏ |
| Use adult tools (e.g., hammers, saws, drills) at home? | ❏ | ❏ | ❏ | ❏ | ❏ | ❏ | ❏ | ❏ | ❏ | ❏ |
| Use adult tools (e.g., hammers, saws, drills) at their early childhood centre / school? | ❏ | ❏ | ❏ | ❏ | ❏ | ❏ | ❏ | ❏ | ❏ | ❏ |
| Roam their neighbourhood with friends but unsupervised by adults? | ❏ | ❏ | ❏ | ❏ | ❏ | ❏ | ❏ | ❏ | ❏ | ❏ |
| Roam their neighbourhood alone? | ❏ | ❏ | ❏ | ❏ | ❏ | ❏ | ❏ | ❏ | ❏ | ❏ |
| Roam their early childhood centre / school grounds during recess and lunch breaks unsupervised by teachers? | ❏ | ❏ | ❏ | ❏ | ❏ | ❏ | ❏ | ❏ | ❏ | ❏ |
| Use loose parts (e.g., sticks, tires, timber, tarpaulins) during outdoor play at home? | ❏ | ❏ | ❏ | ❏ | ❏ | ❏ | ❏ | ❏ | ❏ | ❏ |
| Use loose parts (e.g., sticks, tires, timber, tarpaulins) during outdoor play at their early childhood centre / school? | ❏ | ❏ | ❏ | ❏ | ❏ | ❏ | ❏ | ❏ | ❏ | ❏ |
| Engage in ‘messy’ play (e.g., mud, dirt, sand, water, paint) at home? | ❏ | ❏ | ❏ | ❏ | ❏ | ❏ | ❏ | ❏ | ❏ | ❏ |
| Engage in ‘messy’ play (e.g., mud, dirt, sand, water, paint) at their early childhood centre / school? | ❏ | ❏ | ❏ | ❏ | ❏ | ❏ | ❏ | ❏ | ❏ | ❏ |
| Ride non-motorised vehicles (e.g., bikes, scooters, go-karts) in their neighbourhood while supervised by adults? | ❏ | ❏ | ❏ | ❏ | ❏ | ❏ | ❏ | ❏ | ❏ | ❏ |
| Ride non-motorised vehicles (e.g., bikes, scooters, go-karts) in their neighbourhood with friends but unsupervised by adults? | ❏ | ❏ | ❏ | ❏ | ❏ | ❏ | ❏ | ❏ | ❏ | ❏ |
| Ride non-motorised vehicles (e.g., bikes, scooters, go-karts) in their neighbourhood alone? | ❏ | ❏ | ❏ | ❏ | ❏ | ❏ | ❏ | ❏ | ❏ | ❏ |
| Ride non-motorised vehicles (e.g., bikes, scooters, go-karts) at their early childhood centre / school? | ❏ | ❏ | ❏ | ❏ | ❏ | ❏ | ❏ | ❏ | ❏ | ❏ |
| Climb and play on playground equipment (e.g., monkey bars, ladders, slides)? | ❏ | ❏ | ❏ | ❏ | ❏ | ❏ | ❏ | ❏ | ❏ | ❏ |

**Q6**

Please indicate your level of agreement with the following statements.

|  | Strongly disagree | Disagree | Neither agree nor disagree | Agree | Strongly agree |
| --- | --- | --- | --- | --- | --- |
| Finding ways to get children active is expensive these days. | ❏ | ❏ | ❏ | ❏ | ❏ |
| There are too many unnecessary safety rules applied to children’s play in New Zealand these days. | ❏ | ❏ | ❏ | ❏ | ❏ |
| There are too many unnecessary safety rules in New Zealand schools. | ❏ | ❏ | ❏ | ❏ | ❏ |
| Relaxing the safety rules and introducing traditional ‘risky’ play practices and equipment in schools would enhance childrens development. | ❏ | ❏ | ❏ | ❏ | ❏ |
| Relaxing the safety rules and introducing traditional ‘risky’ play practices and equipment in schools would result in an increase in serious accidents and injuries at school. | ❏ | ❏ | ❏ | ❏ | ❏ |
| Children require regular exposure to actual risk in order to develop risk management skills. | ❏ | ❏ | ❏ | ❏ | ❏ |

**Q7**

Please express your opinion about the following statements regarding 9- or 10-year-olds (boys or girls) who go out alone in the area around your home.I believe that a 9- 10-year-old boy or girl who goes out alone in the area around my house can:

|  | Unlikely | Not very likely | Likely | Very likely |
| --- | --- | --- | --- | --- |
| Make new friends | ❏ | ❏ | ❏ | ❏ |
| Be exposed to the risk of road accidents | ❏ | ❏ | ❏ | ❏ |
| Encounter ill-intentioned adults | ❏ | ❏ | ❏ | ❏ |
| Learn his/her way around | ❏ | ❏ | ❏ | ❏ |
| Meet and/or play with other children | ❏ | ❏ | ❏ | ❏ |
| See things that may frighten her/him | ❏ | ❏ | ❏ | ❏ |
| Become more responsible | ❏ | ❏ | ❏ | ❏ |
| Find someone willing to help him/her in case of trouble | ❏ | ❏ | ❏ | ❏ |
| Feel disoriented when among people | ❏ | ❏ | ❏ | ❏ |

**SECTION 3**

Child Activities

**Q8:**

Is there at least one dependent child aged 5-12 years that currently lives in your household most nights of the week?

1. Yes
2. No

The questions in this section refer to the oldest dependent child aged 5-12 years that currently lives in your household most nights of the week.

**Q9:**

What is the age of your child?

1. 5 years
2. 6 years
3. 7 years
4. 8 years
5. 9 years
6. 10 years
7. 11 years
8. 12 years

**Q10:**

What is your relationship to your child?

1. Mother
2. Father
3. Step-mother
4. Step-father
5. Grandmother
6. Grandfather
7. Aunt
8. Uncle
9. Guardian
10. Other

**Q11:**

Does your child have an intellectual or physical disability?

1. Yes
2. No

**SECTION 4**

**Q12:**

The next questions in this section refer to the rules you apply to your oldest dependent child aged 5-12 years that currently lives in your household most nights of the week.

Please answer yes or no to the following questions.

|  | Yes | No |
| --- | --- | --- |
| Would you let the child jump down from a height of 3–4 m? | ❏ | ❏ |
| Would you allow the child to play chase with other children? | ❏ | ❏ |
| Would you trust the child to play by themselves without constant supervision? | ❏ | ❏ |
| Would you let the child go down head first down a slippery dip? | ❏ | ❏ |
| Would you allow the child to continue playing if they get a few scrapes during play? | ❏ | ❏ |
| Would you let the child have lots of challenges when they play at home? | ❏ | ❏ |
| Would you let the child use a hammer and nail unsupervised? | ❏ | ❏ |
| Would you let the child climb up a tree within your reach? | ❏ | ❏ |
| Would you let the child walk barefoot across a floor after broken glass had been swept up? | ❏ | ❏ |
| Would you let the child walk on slippery rocks close to water? | ❏ | ❏ |
| Would you allow the child to play-fight other children with sticks? | ❏ | ❏ |
| Would you encourage the child to try new things that involve some risk? | ❏ | ❏ |
| Would you allow the child to engage in rough and tumble play? | ❏ | ❏ |
| Would you let the child play near the edge of steep cliffs? | ❏ | ❏ |
| Would you allow the child to play in the bush out of your sight? | ❏ | ❏ |
| Would you let the child experience minor mishaps if what they are doing is lots of fun? | ❏ | ❏ |
| Would you let the child run close to an open fire? | ❏ | ❏ |
| Would you let the child swim in the ocean close to the shore while you were watching from the beach? | ❏ | ❏ |
| Would you allow the child to continue playing if there is the potential they may break a bone? | ❏ | ❏ |
| Would you let the child play in a backyard unsupervised? | ❏ | ❏ |
| Would you allow the child to play-fight, testing who is strongest? | ❏ | ❏ |
| Would you allow the child to climb a rock wall that goes straight down to the water? | ❏ | ❏ |
| Would you wait to see if the child could manage challenges on their own before getting involved? | ❏ | ❏ |
| Would you let the child climb as high as they want to in trees? | ❏ | ❏ |
| Would you allow the child to ride a bicycle down a steep hill at full speed? | ❏ | ❏ |
| Would you trust the child to play safely? | ❏ | ❏ |
| Would you allow the child to use a sharp knife? | ❏ | ❏ |
| Would you let the child play in a backyard supervised? | ❏ | ❏ |
| Would you let the child balance on a fallen tree more than 2m above the ground? | ❏ | ❏ |
| Would you encourage the child to take some risks if it means having fun during play? | ❏ | ❏ |
| Would you allow the child to climb up a tree beyond your reach? | ❏ | ❏ |

**SECTION 5**

**Q13:**

The next question in this section refers to the activity patterns of your oldest dependent child aged 5-12 years that currently lives in your household most nights of the week.

How often does your child engage in the following activities?

|  | Never | Seldom | Sometimes | Often | Always | N/A |
| --- | --- | --- | --- | --- | --- | --- |
| Use adult tools (e.g., hammers, saws, drills) unsupervised. | ❏ | ❏ | ❏ | ❏ | ❏ | ❏ |
| Climb trees. | ❏ | ❏ | ❏ | ❏ | ❏ | ❏ |
| Engage in rough-and-tumble games (e.g., wrestling, bullrush). | ❏ | ❏ | ❏ | ❏ | ❏ | ❏ |
| Roam their neighbourhood with friends but unsupervised by adults. | ❏ | ❏ | ❏ | ❏ | ❏ | ❏ |
| Roam their neighbourhood alone. | ❏ | ❏ | ❏ | ❏ | ❏ | ❏ |
| Use loose parts (e.g., sticks, tires, timber, tarpaulins) when they play outdoors. | ❏ | ❏ | ❏ | ❏ | ❏ | ❏ |
| Engage in ‘messy’ play (e.g., mud, dirt, sand, water, paint). | ❏ | ❏ | ❏ | ❏ | ❏ | ❏ |
| Ride non-motorised vehicles (e.g., bikes, scooters, go-karts) in the neighbourhood while supervised by adults. | ❏ | ❏ | ❏ | ❏ | ❏ | ❏ |
| Ride non-motorised vehicles (e.g., bikes, scooters, go-karts) in the neighbourhood with friends but unsupervised by adults. | ❏ | ❏ | ❏ | ❏ | ❏ | ❏ |
| Ride non-motorised vehicles (e.g., bikes, scooters, go-karts) in the neighbourhood alone. | ❏ | ❏ | ❏ | ❏ | ❏ | ❏ |

**Q14:**

How many days in the last seven days did your child do the following activities?

|  | No days | One day | Two days | Three days | Four days | Five days | Six days | Seven days |
| --- | --- | --- | --- | --- | --- | --- | --- | --- |
| Play or practice organised sport. | ❏ | ❏ | ❏ | ❏ | ❏ | ❏ | ❏ | ❏ |
| Participate in other organised or structured activities (e.g., dance, gym, music lessons). | ❏ | ❏ | ❏ | ❏ | ❏ | ❏ | ❏ | ❏ |

**Q15:**

How often do you allow your child to play outside when it is raining?

1. Never
2. Rarely
3. Sometimes
4. Often
5. Always

**Q16:**

What are some of the reasons that might make you decide not to allow your child to play outside in the rain? You may choose more than one option.

1. It will be too cold for my child
2. My child may get sick
3. My child will get too messy
4. My child may slip or have an accident
5. I dont like being outside in the rain
6. My child doesnt like being outside in the rain
7. My child does not have suitable weatherproof clothing (e.g., raincoats)
8. I do not have suitable weatherproof clothing (e.g., raincoats)
9. Other

**SECTION 6**

:

The next question in this section refer to the values you apply to your oldest dependent child aged 5-12 years that currently lives in your household most nights of the week.

**Q17:**

Please indicate your level of agreement with the following statements.

|  | Very strongly disagree | Strongly disagree | Disagree | Neither agree nor disagree | Agree | Strongly agree | Very strongly agree |
| --- | --- | --- | --- | --- | --- | --- | --- |
| I am concerned about the things I cannot control that can physically injure my child. | ❏ | ❏ | ❏ | ❏ | ❏ | ❏ | ❏ |
| Fewer injuries happen to children when parents plan ways to prevent them. | ❏ | ❏ | ❏ | ❏ | ❏ | ❏ | ❏ |
| I am concerned about the potential hazards in my home. | ❏ | ❏ | ❏ | ❏ | ❏ | ❏ | ❏ |
| Children should play in places where there is low risk of injury. | ❏ | ❏ | ❏ | ❏ | ❏ | ❏ | ❏ |
| Good supervision of my child means knowing what my child is doing at all times. | ❏ | ❏ | ❏ | ❏ | ❏ | ❏ | ❏ |
| Letting my child engage in physical activities without supervision greatly increases their chance of injury. | ❏ | ❏ | ❏ | ❏ | ❏ | ❏ | ❏ |
| It is important for my child to engage in physically challenging experiences. | ❏ | ❏ | ❏ | ❏ | ❏ | ❏ | ❏ |
| I like to let my child find his or her own physical limits. | ❏ | ❏ | ❏ | ❏ | ❏ | ❏ | ❏ |
| I value opportunities for my child to explore new environments. | ❏ | ❏ | ❏ | ❏ | ❏ | ❏ | ❏ |
| Benefits of physical activity for my child outweigh the risk of experiencing minor injuries. | ❏ | ❏ | ❏ | ❏ | ❏ | ❏ | ❏ |
| I prefer to teach my child how to manage risky situations rather than avoid them. | ❏ | ❏ | ❏ | ❏ | ❏ | ❏ | ❏ |
| Participating in challenging and potentially risky physical activities will help my child develop self-confidence. | ❏ | ❏ | ❏ | ❏ | ❏ | ❏ | ❏ |
| It feels as if I am always driving my child to an organised sport or activity. | ❏ | ❏ | ❏ | ❏ | ❏ | ❏ | ❏ |
| The number of organised sports or activities my child participates in is a source of stress for the family. | ❏ | ❏ | ❏ | ❏ | ❏ | ❏ | ❏ |

**SECTION 7**

The next questions in this section refer to the travel patterns of your oldest dependent child aged 5-12 years that currently lives in your household most nights of the week.

**Q18:**

Does your child currently attend a primary or intermediate school?

1. Yes
2. No

**Q19:**

How does your child usually travel to school? You may select more than one option.

1. Walk
2. Car
3. Bicycle/Scooter
4. Bus
5. Other

**Q20:**

Who does your child usually travel to school with? You may select more than one option.

1. On their own
2. Adult(s)
3. Sibling(s)
4. Friend(s)
5. Other

**Q21:**

How does your child usually travel from school? You may select more than one option.

1. Walk
2. Car
3. Bicycle/Scooter
4. Bus
5. Other

**Q22:**

Who does your child usually travel from school with? You may select more than one option.

1. On their own
2. Adult(s)
3. Sibling(s)
4. Friend(s)
5. Other

**Q23:**

Please answer yes or no to the following questions.

|  | Yes | No | N/A |
| --- | --- | --- | --- |
| Does your child travel home from school alone? | ❏ | ❏ | ❏ |
| When going to places other than school that are within walking distance, is your child allowed to go alone? | ❏ | ❏ | ❏ |
| Is your child allowed to cross main roads alone? | ❏ | ❏ | ❏ |
| Is your child usually allowed to go out alone after dark? | ❏ | ❏ | ❏ |
| Is your child allowed to cycle on main roads alone? | ❏ | ❏ | ❏ |
| Is your child usually allowed to travel on local buses alone (other than a school bus)? | ❏ | ❏ | ❏ |

**SECTION 8**

The next question in this section refers to the screen time patterns of your oldest dependent child aged 5-12 years that currently lives in your household most nights of the week. Screen time refers to any time spent in front of the television, computer, gaming console (Playstation, Xbox, Wii), tablet (iPad), smartphone, or any other electronic equipment.

**Q24:**

In the last seven days, how much time did your child spend on a screen:

|  | Less than one hour | Between one and two hours | Between two and three hours | Between three and four hours | More than four hours | N/A |
| --- | --- | --- | --- | --- | --- | --- |
| On an average weekday (Mon-Fri) for recreational purposes? | ❏ | ❏ | ❏ | ❏ | ❏ | ❏ |
| On an average weekend day (Sat-Sun) for recreational purposes? | ❏ | ❏ | ❏ | ❏ | ❏ | ❏ |
| On an average weekday (Mon-Fri) for homework or work purposes? | ❏ | ❏ | ❏ | ❏ | ❏ | ❏ |
| On an average weekend day (Sat-Sun) for homework or work purposes? | ❏ | ❏ | ❏ | ❏ | ❏ | ❏ |

**Q25:**

How often do you allow your child to watch television or use an electronic device at dinner time?

1. Never
2. Rarely
3. Sometimes
4. Often
5. Always

**Q25a:**

How often do you enroll your child in a supervised school holiday programme?

1. Never
2. Rarely
3. Sometimes
4. Often
5. Always

Family Dynamics

**Q26:**

Please indicate if the following statements are mostly true or mostly false.

|  | Mostly true | Mostly false | Dont know |
| --- | --- | --- | --- |
| Family members really help and support one another. | ❏ | ❏ | ❏ |
| We often seem to be killing time at home. | ❏ | ❏ | ❏ |
| We put a lot of energy into what we do at home. | ❏ | ❏ | ❏ |
| There is a feeling of togetherness in our family | ❏ | ❏ | ❏ |
| We rarely volunteer when something has to be done at home. | ❏ | ❏ | ❏ |
| Family members really back each other up. | ❏ | ❏ | ❏ |
| There is little group spirit in our family. | ❏ | ❏ | ❏ |
| We get along really well with each other. | ❏ | ❏ | ❏ |
| There is little time and attention for everyone in the family. | ❏ | ❏ | ❏ |

Wellbeing

**Q27:**

|  | 0 - Extremely unhappy | 1 | 2 | 3 | 4 | 5 | 6 | 7 | 8 | 9 | 10 - Extremely happy |
| --- | --- | --- | --- | --- | --- | --- | --- | --- | --- | --- | --- |
| Taking all things together, how happy would you say you are on a scale of 0 to 10? | ❏ | ❏ | ❏ | ❏ | ❏ | ❏ | ❏ | ❏ | ❏ | ❏ | ❏ |

**Q28:**

|  | 0 - Extremely dissatisfied | 1 | 2 | 3 | 4 | 5 | 6 | 7 | 8 | 9 | 10 - Extremely satisfied |
| --- | --- | --- | --- | --- | --- | --- | --- | --- | --- | --- | --- |
| All things considered, how satisfied are you with your life as a whole nowadays on a scale from 9 to 10? | ❏ | ❏ | ❏ | ❏ | ❏ | ❏ | ❏ | ❏ | ❏ | ❏ | ❏ |

**Q29:**

|  | 0 - None of the time | 1 | 2 | 3 | 4 | 5 | 6 | 7 | 8 | 9 | 10 - All of the time |
| --- | --- | --- | --- | --- | --- | --- | --- | --- | --- | --- | --- |
| How much of the time would you generally say you are enthusiastic about what you are doing on a scale from 0 to 10? | ❏ | ❏ | ❏ | ❏ | ❏ | ❏ | ❏ | ❏ | ❏ | ❏ | ❏ |

**SECTION 9**

Demographics

**Q30a:**

What is your gender?

1.Male

2.Female

**Q30:**

What is your age?

1. Younger than 25 years
2. 25-29 years
3. 30-34 years
4. 35-39 years
5. 40-44 years
6. 45-49 years
7. 50-54 years
8. 55-59 years
9. 60 years or older

**Q31:**

What ethnic group(s) do you belong to?

1. New Zealand European
2. New Zealand Maori
3. Samoan
4. Cook Island Maori
5. Tongan
6. Niuean
7. Chinese
8. Indian
9. Korean
10. Other

**Q32:**

Where in New Zealand do you usually live?

1. Northland
2. Auckland
3. Waikato
4. Bay of Plenty
5. Gisbourne
6. Hawkes Bay
7. Taranaki
8. Manawatu-Whanganui
9. Wellington
10. Tasman
11. Marlborough
12. West Coast
13. Canterbury
14. Otago
15. Southland
16. Other

**Q33:**

What location best describes where you live?

1. Large city (more than 100,000 people)
2. Smaller city (30,000 to 100,000 people)
3. Town (1,000 to 29,999 people)
4. Small town, community, or village (less than 1,000 people)
5. Rural (not a small town)
6. Other

**Q34:**

What is the highest academic qualification you have completed?

1. Finished primary school
2. Finished secondary school
3. University Entrance / Bursary / Scholarship (or equivalent)
4. Apprenticeship, diploma, trade certificate
5. Bachelor degree
6. Postgraduate diploma / degree or higher
7. Prefer not to answer
8. Other

**Q35:**

What is your annual household income?

1. Zero income
2. $1 – $20,000
3. $20,001 – $40,000
4. $40,001 – $60,000
5. $60,001 – $80,000
6. $80,001 – $100,000
7. $100,001 – $150,000
8. $150,001 or more
9. Prefer not to answer

**Q36:**

What best describes you current employment situation?

1. Working in paid employment - or away temporarily
2. Not in paid work and looking for a job
3. In education - or on holiday
4. Permanently sick or disabled
5. Retired
6. Doing housework, looking after children or other persons
7. Prefer not to answer
8. Other

**Q37:**

Are you currently on maternity or paternity leave?

1. Yes
2. No

1. State of Play Survey 2015
